# Clinical analysis of subxiphoid single-port thoracoscopic surgery for simultaneous bilateral lung lesion resection

**DOI:** 10.1186/s12893-022-01646-4

**Published:** 2022-05-25

**Authors:** Jun Wang, Meiqing Xu, Chuankai Zhang, Dazhong Wei

**Affiliations:** Department of Thoracic Surgery, The First Affiliated Hospital of University of Science and Technology of China, Hefei, 230001 People’s Republic of China

**Keywords:** Subxiphoid, Intercostal, Single-port thoracoscopic, Bilateral lung lesions, Simultaneous

## Abstract

**Objective:**

To investigate the efficacy and safety of simultaneous subxiphoid single-port thoracoscopic resection of bilateral lung lesions.

**Methods:**

This retrospective study analyzed the clinical data of 72 patients who underwent resection of bilateral lung lesions at the Department of Thoracic Surgery in the First Affiliated Hospital of University of Science and Technology of China between August 2020 and January 2022. Surgery-related parameters were compared between patients who underwent subxiphoid single-port thoracoscopy (subxiphoid group, 36 patients) and patients who underwent intercostal single-port thoracoscopy (intercostal group, 36 patients).

**Results:**

Compared to the intercostal group, the subxiphoid group exhibited significantly better postoperative catheterization time (*P* = 0.013), postoperative thoracic drainage, postoperative visual analog scale pain scores at 24 and 48 h, and incision pain and numbness at 1 and 3 months after surgery (all *P* < 0.05). There were no significant differences in operation time, intraoperative blood loss, or postoperative complications between the two groups (all *P* > 0.05). There were no cases of perioperative mortality, conversion to thoracotomy, or serious complications in either group.

**Conclusion:**

Subxiphoid single-port thoracoscopic surgery for simultaneous resection of bilateral lung lesions is safe and effective, reduces postoperative acute and chronic pain, decreases trauma, allows faster recovery, and is more consistent with the concept of minimally invasive surgery than bilateral intercostal single-port thoracoscopy. Thus, this subxiphoid single-port thoracoscopic surgery approach should be considered for clinical application.

## Introduction

Subxiphoid single-port thoracoscopic surgery has been increasingly used for the treatment of pulmonary diseases, mainly in cases that involve lesions in a single lung. However, there have been few reports of subxiphoid surgery for the resection of bilateral lung lesions. To explore the effectiveness of this operation, 72 patients with bilateral lung lesions underwent single-port thoracoscopic surgery via subxiphoid or intercostal approaches between August 2020 and January 2022. Simultaneous bilateral pulmonary lesion resection was performed in the same manner, and the results were compared between the two groups.

## Materials and methods

### General information

This retrospective study analyzed the clinical data of 72 patients who underwent resection of bilateral lung lesions at the Department of Thoracic Surgery in the First Affiliated Hospital of University of Science and Technology of China between August 2020 and January 2022. Comparisons were performed between patients who underwent subxiphoid single-port thoracoscopy (subxiphoid group, 36 patients) and patients who underwent bilateral intercostal single-port thoracoscopy (intercostal group, 36 patients). 72 cases were randomly divided into the two groups, all cases strictly followed the inclusion and exclusion criteria.

The inclusion criteria were: lesions in both lungs that preoperative computed tomography (CT) confirmed to be peripheral pure ground-glass or mixed ground-glass nodules ≤ 2 cm in diameter, with suitability for wedge-shaped resection; lesion identification as atypical adenomatous hyperplasia, adenoma in situ, or minimally invasive cancer on intraoperative frozen section analysis; peripheral lesions ≤ 4 cm in diameter on CT that were identified as invasive lung cancer on intraoperative frozen section analysis, but in patients with poor cardiopulmonary function or other serious complications who underwent compromised lung wedge resection; peripheral lesions ≤ 4 cm in diameter on CT that were identified as benign lung tumors (e.g., sclerosing hemangioma, inflammatory pseudotumor, or hamartoma) on intraoperative frozen section analysis. Approval was obtained from the hospital ethics committee and all patients provided written informed consent.

The exclusion criteria were: preoperative CT evidence of central lesions or lung lesions > 4 cm in diameter unsuitable for wedge-shaped resection, intraoperative frozen section evidence of invasive cancer requiring segmentectomy or lobectomy, lesion location outside of the lower left lung and posterior basal segment, history of lung surgery, history of pulmonary tuberculosis and pleuritis, substernal angle outside the normal range (< 70°), body mass index > 30 kg/m^2^, complications of cardiac diseases (e.g., arrhythmia or cardiac hypertrophy), and lack of consent.

### Surgical methods

All patients in both groups underwent preoperative examinations, including thin-slice CT and three-dimensional reconstruction of the lungs; they were screened in strict accordance with the inclusion and exclusion criteria outlined above. Preoperative CT-guided localization was performed for ground-glass nodules < 2 cm in diameter.

**Fig. 1:**
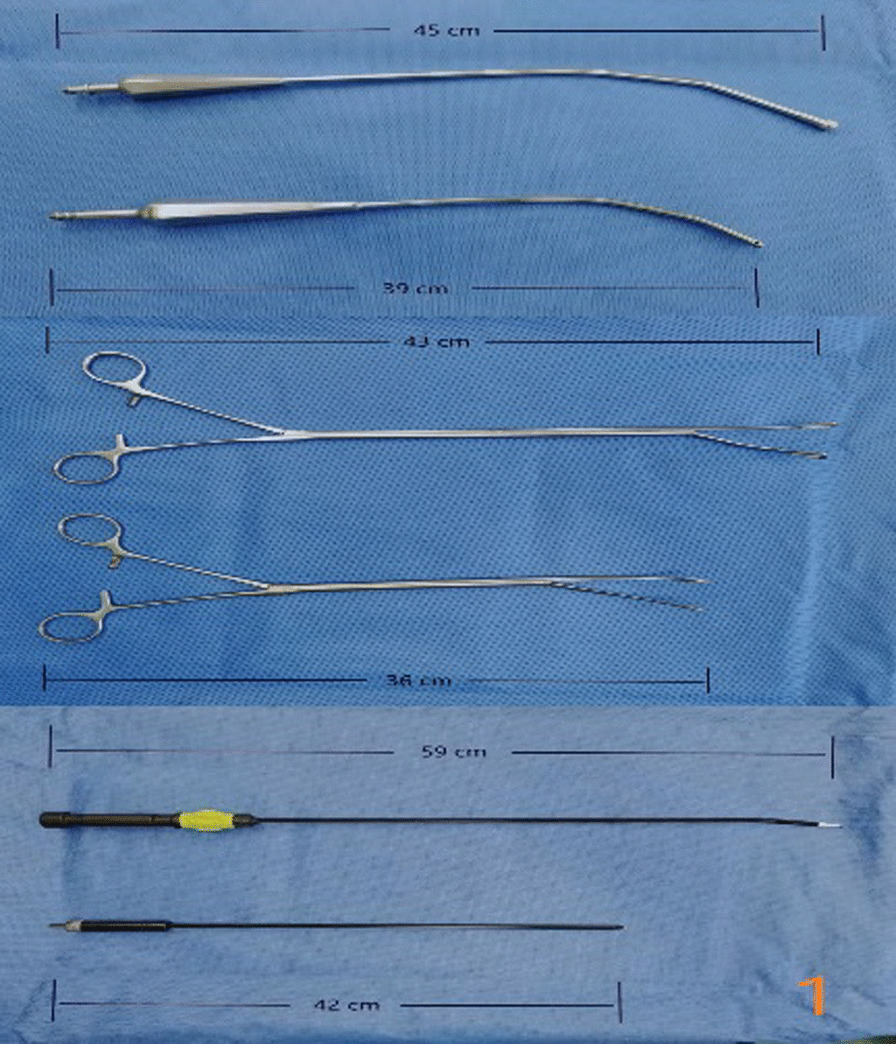
Comparison of subxiphoid thoracoscopic instruments and intercostal thoracoscopic instruments

**Fig. 2:**
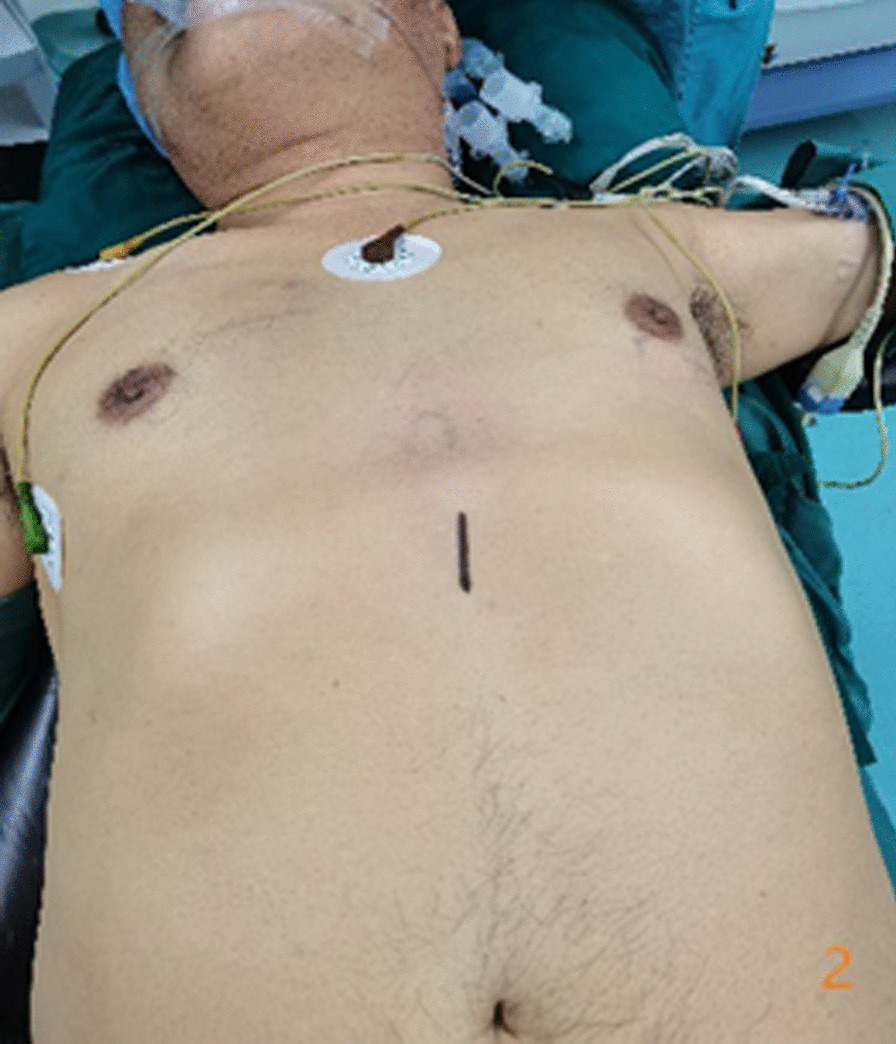
Incision location

**Fig. 3:**
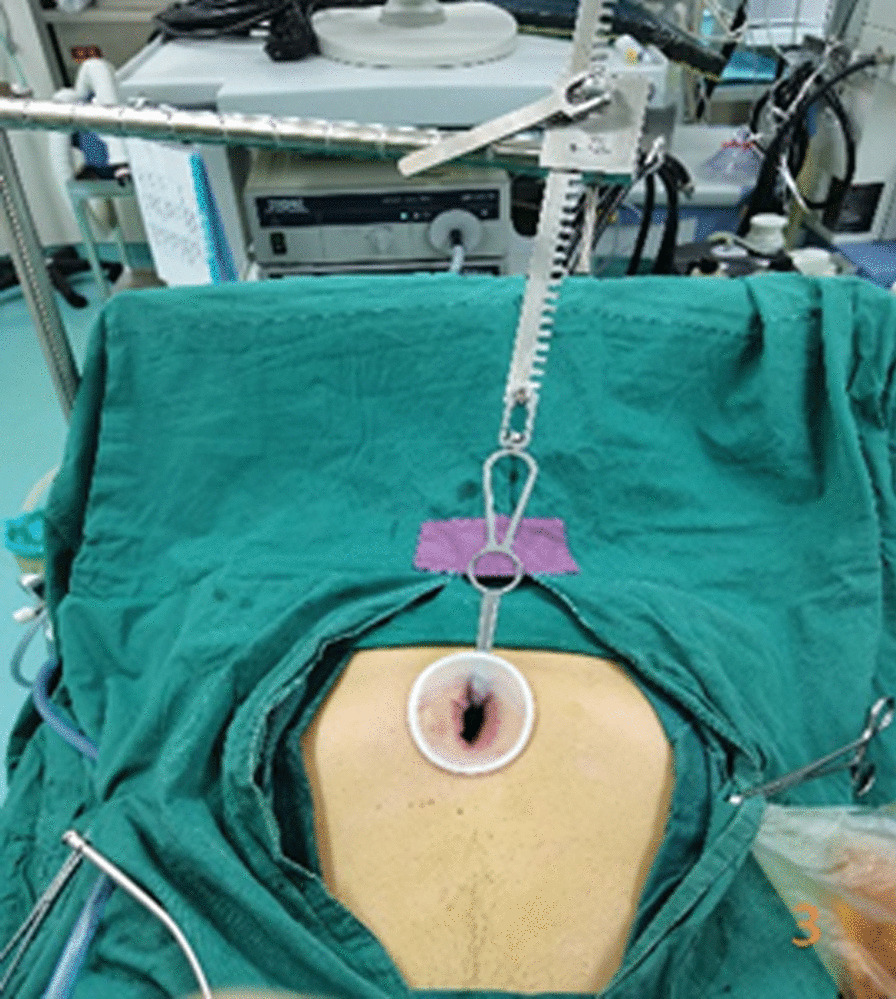
Using the sternum retractor and incision protector

**Fig. 4:**
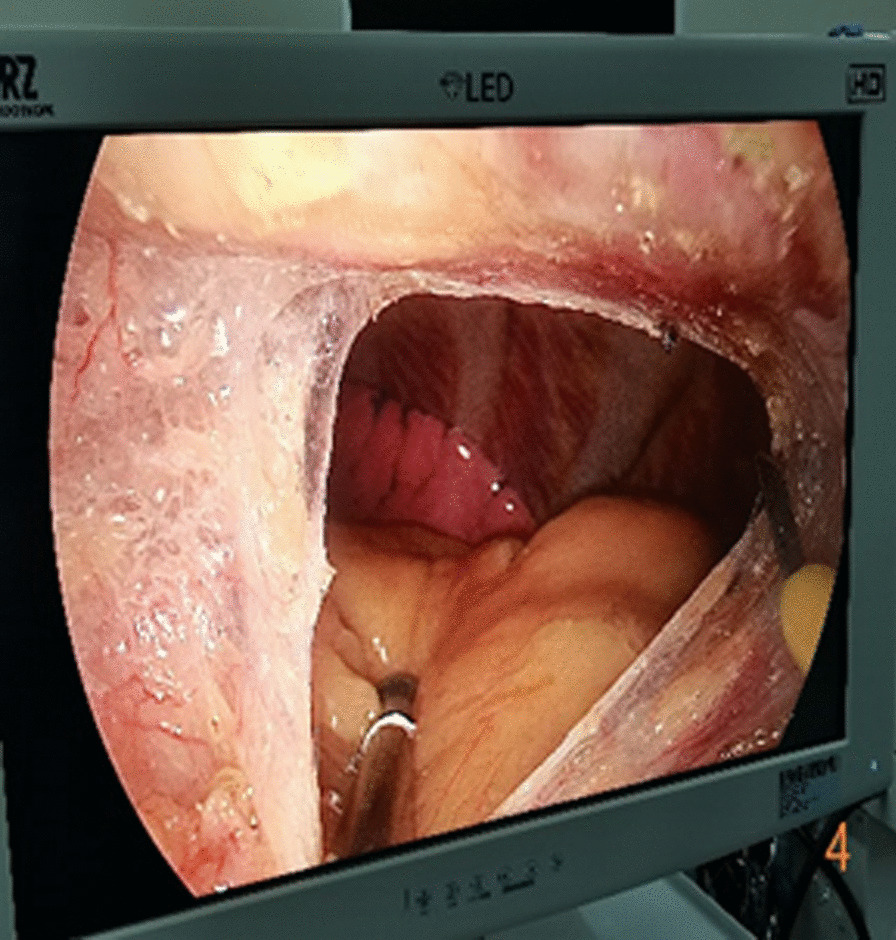
Puncture the pleura into the chest cavity

**Fig. 5:**
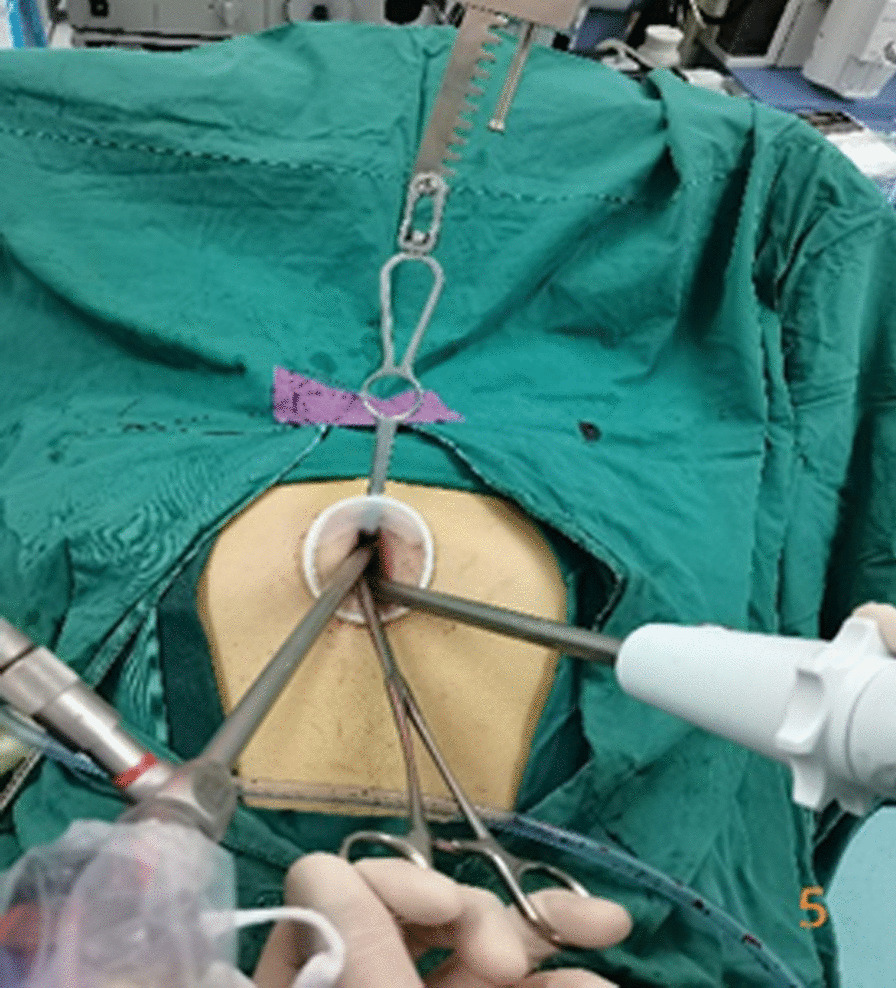
Device interaction

**Fig. 6:**
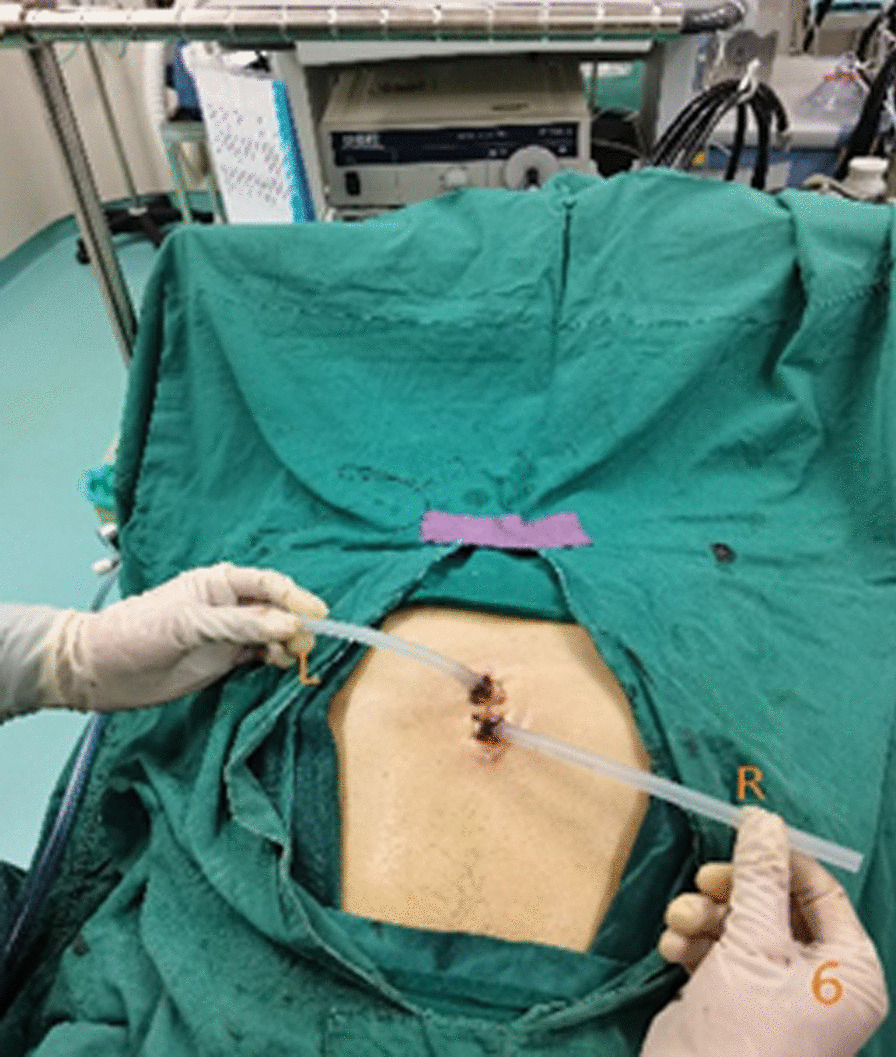
Placing a chest tube

In the subxiphoid group, the patient was placed in the supine position with the back raised and the hands naturally on both sides of the body to fully expose the subxiphoid, chest, and upper abdomen. The height of the operating table was adjusted for the surgeon, and a longitudinal incision ~ 3 cm in length was made below the xiphoid process in a layer-by-layer manner to the level of the xiphoid process. The larger xiphoid process was bluntly excised close to the retrosternal space, leaving a gap of 3–5 cm. A sternum retractor was used to enhance the retrosternal space, and a 30° thoracoscope was placed in the gap using an incision protection sleeve. Extended suction, oval forceps, and other instruments were used intraoperatively as necessary. After entry into one side of the thoracic cavity, a linear cutting suture was used to perform lung wedge resection; the lung lesions were completely removed. The lesion was confirmed to be > 2 cm from the edge of the incision, For solid tumors > 3 cm, due to the wider base, the wedge-shaped lung resection range is large, and the lung resection margin is intermittently sutured to prevent bleeding and air leakage after the lung resection margin;the specimens were then placed in a specimen bag and removed. After confirmation of no air leakage at the incision margin, the other side of the thoracic cavity was re-entered; the other side of the lung lesion was resected in the same manner. During the operation, depending on the exposure of the operative field, the operating table could be rotated to both sides, and the chest catheters on both sides were drawn through the single-hole incision under the xiphoid process, as shown in Figs. 1, 2, 3, 4, 5, 6.

In the intercostal group, the operation was performed with the patient lying on one side with the upper limb elevated, stretched forward, and fixed in position. A single incision ~ 3 cm in length was made in the fifth intercostal space of the anterior axillary line. The operation was performed in the same manner as in the subxiphoid group. After the operation on one side, an indwelling chest catheter was placed, and the incision was sutured in a layer-by-layer manner. Wedge resection surgery was performed as described above.

Patient-controlled analgesia was used routinely in both groups after the operation. Patients were administered prophylactic antibiotics for 24 h; they were encouraged to commence early activity along with coughing and expectoration. Postoperative chest X-rays showed good recruitment of both lungs. The daily drainage volume of the thoracic drainage tube was < 150 mL, there was no air leakage within 24 h, and the body temperature remained normal in all patients. Both thoracic drainage tubes were removed concurrently or in stages according to the recovery of both thoracic cavities.

Pain was scored using a 10-point visual analog scale as follows: 0, no pain; ≤ 3, mild pain, tolerable, does not affect rest; 4–6, pain affects rest, requires analgesia; 7–10, unbearable pain, affects both appetite and sleep.

### Indicators

The operation time, intraoperative blood loss, postoperative catheterization time, total chest tube drainage volume, postoperative incision pain score, postoperative complications, and long-term incision pain or numbness were recorded in the two groups.

### Statistical analysis

Statistical analyses were performed using SPSS 21.0 (SPSS Inc., Chicago, IL, USA). The measurement data were first tested for normality of the distribution. Continuous data with a normal distribution are expressed means ± standard deviations, and the *t* test was used for comparisons between groups. Continuous data with a non-normal distribution are expressed as medians (interquartile ranges), and the Mann–Whitney U test was used for comparisons between groups. Categorical data are expressed as numbers and percentages, and comparisons between groups were performed by the χ^2^ test. In all analyses, *P* < 0.05 was considered to indicate statistical significance.

## Results

There were no significant differences in sex distribution, age, or postoperative pathological results between the two groups (*P* > 0.05) (Table 1).

**Table 1:** Comparison of general data

	Xiphoid group (n = 36)	Intercostal group (n = 36)	Statistical value	P value
Sex (n)				
Male	15	20	χ^2^ = 1.390	0.238
Female	21	16		
Age (years, x ± s)	44.333 ± 15.059	46.305 ± 14.585	t = 0.564	0.574
Postoperative pathology atypical adenomatous hyperplasia (n)	5	6	χ^2^ = 0.790*	0.972
Carcinoma in situ (n)	14	15		
Minimally invasive adenocarcinoma (n)	9	7		
Adenocarcinoma (n)	3	4		
Benign lesions (n)	5	4		

*Fisher’s exact probability method

Compared to the intercostal group, the subxiphoid single-port thoracoscopy group exhibited significantly better postoperative catheter placement time (*P* = 0.013), total thoracic drainage volume (*P* = 0.018), visual analog scale pain scores at 24 and 48 h after the operation (*P* < 0.000 and *P* < 0.000), and incision pain and numbness at 1 and 3 months after the operation (*P* = 0.006 and *P* = 0.003). There were no significant differences in operation time, intraoperative blood loss, or postoperative complications between the two groups (all *P* > 0.05) (Table 2).

**Table 2:** Comparison of intraoperative and postoperative data

	Xiphoid group (n = 36)	Intercostal group (n = 36)	Statistical value	P value
Operative time (min, x ± s)	66.972 ± 13.246	64.139 ± 15.262	t = 0.841	0.403
Intraoperative blood loss (ml, x ± s)	51.528 ± 17.921	49.722 ± 19.159	t = 0.413	0.681
Intubation time (d)	3.0(2.0,4.0)	3.0(3.0,5.0)	U = 436.500*	0.013
total thoracic drainage(ml, x ± s)	261.944 ± 69.850	308.611 ± 91.843	t = 2.427	0.018
complications (n)	2	2	χ^2^ = 0.000**	1.000
Arrhythmia	0	2		
Prolonged air leak	1	0		
Incision fat liquefaction	1	0		
Postoperative pain score (VAS scale)				
24 h after surgery	2.667 ± 0.926	4.750 ± 1.628	t = 6.675	< 0.000
48 h after surgery	2.917 ± 1.251	4.389 ± 1.591	t = 4.365	< 0.000
Discharge follow-up for pain or numbness in the incision				
1 month after surgery	2	11	χ^2^ = 7.604	0.006
3 month after surgery	1	10	χ^2^ = 8.692	0.003

The operation time of the control group included the time when the body position was changed again during the operation to disinfect the towel

*Indicates sampling nonparametric test (Mann–Whitney U); **Fisher exact probability method

There were no cases of perioperative mortality, conversion to thoracotomy, or increased surgical incision, and no serious complications (e.g., intrathoracic hemorrhage or pulmonary embolism) after the operation throughout the study population. In the subxiphoid group, one case of postoperative pulmonary air leakage was resolved after intrathoracic injection of erythromycin, and one case of incision fat liquefaction was resolved by intensive incision dressing changes. In the intercostal group, there were two cases of arrhythmia after the operation: atrial fibrillation and atrial premature beat (one case each). All patients were discharged after successful treatment of symptoms.

### Comments

Traditional intercostal single-port thoracoscopic treatment of pulmonary lesions has the advantage of reduced trauma, rapid recovery, and high patient satisfaction [1, 2]. However, for thoracoscopic surgery to concurrently remove lesions in both lungs, it remains necessary to enter the thoracic cavity through both sides of the intercostal space for surgery. Regardless of whether both sides are treated using single-port thoracoscopic surgery, two surgical incisions are required; this inevitably causes damage to the intercostal nerves on both sides. In addition, the body position must be changed and the drape must be re-sterilized during the operation, thus increasing both the use of surgical consumables and the corresponding amounts of time and effort. With the development of minimally invasive thoracoscopic techniques, there has been considerable interest in identifying a less invasive surgical approach for the simultaneous resection of bilateral lung lesions [3–5]. Many thoracic surgeons have attempted subxiphoid single-port thoracoscopic lobectomy since it was first reported in 2014 by Liu et al. [6]. There have been reports of single-port thoracoscopic resection of single-lung lesions, but there have been few reports of simultaneous resection of double-lung lesions; the efficacy and safety of the surgery have remained uncertain.

The subxiphoid approach has multiple advantages in comparison with the bilateral intercostal approach to resection of bilateral lung lesions. First, the subxiphoid approach avoids damage to the bilateral intercostal nerves, thereby avoiding the potential for acute nerve pain in the thoracic incision. In this study, the pain scores in the subxiphoid group at 24 and 48 h after the operation were lower than the corresponding pain scores in the intercostal group (*P* < 0.05). The relief of pain in the subxiphoid incision enables patients to more easily cough and expectorate after surgery, promotes lung recruitment, and enhances the drainage of pleural effusion; these benefits can lead to early extraction of the chest catheter. The total drainage volume significantly differed between the subxiphoid and intercostal groups in the present study (*P* < 0.05). Second, compared with two chest incisions, one incision under the xiphoid process greatly reduces the occurrence of long-term intractable pain and improves long-term quality of life for patients; consistent with this perspective, we observed significant differences in the incidences of incision pain or numbness at 1 and 3 months after the operation between the two groups (*P* < 0.05). Third, with one incision, only one position is required to remove the bilateral lung lesions. Thus, there is no need to reposition the patient during surgery; this shortens the operation and anesthesia times, while reducing the use of medical consumables. However, we observed no significant difference in the operation time between the two groups in this study (*P* > 0.05), presumably because of the longer learning curve for subxiphoid single-port thoracoscopic surgery than for traditional intercostal single-port thoracoscopic surgery [7–10].

Because of the learning curve, subxiphoid single-port thoracoscopic surgery is carried out only in larger medical centers. There is considerable distance between the surgical incision in the subxiphoid approach and the lung tissue; thus, the surgical path becomes obliquely long and the surgical field of view changes, leading to necessary operator adaptation [11, 12]. Because of cardiac pulsation, the operation is difficult in the left lung; operations involving the outer and posterior basal segments of the left lower lung are generally considered to be contraindicated.

However, the learning curve may be shortened by accumulating more cases and continuously reporting experience with the subxiphoid approach. Longer double-joint instruments with a curved head should be used where possible to facilitate the operation [13]. A sternum retractor should be used to increase the retrosternal space, and an incision protection sleeve should be used to ensure thoracoscopic lens clarity. The lens should be kept at the top of the incision, with 30° oblique illumination of the thoracic cavity from top to bottom, double joint oval forceps and straight cut stapler are used in the operation through the lower end of the incision, which can avoid interference between surgical instruments and the lens [14, 15].

There are still many limitations in this study. It included few cases and the follow-up time was short, so further research is needed for large samples and long-term follow-up. The selection bias can also affect study results. At the same time, the recurrence was not observed in this study. In the future study, the observation time will be extended, various indicators will be improved.

In summary, suitable patient selection and simultaneous surgical resection of bilateral lung lesions with subxiphoid single-port thoracoscopic surgery can reduce the intubation time, relieve acute and chronic incision pain, and improve the long-term safety and efficacy of the operation. This can improve patient quality of life and overall acceptance by patients.

## Data Availability

The datasets used and/or analysed during the current study are available from the corresponding author on reasonable request.
